# Southern Dental Association

**Published:** 1894-09

**Authors:** J. M. Walker


### Southern Dental Association.

Twenty-fifth Annual Meeting, Old Point Comfort, Va., August 2–6, 1894.

Reported for the Dental Register by Mrs. J. M. Walker.

The Southern Dental Association convened in twenty-fifth annual session, at Old Point Comfort, Virginia, August 2, 1894.

The meeting was called to order by the President, Dr. B. Holly Smith, Baltimore, Md., and opened with prayer by Dr. W. H. Morgan, in the absence of the Chaplain of the Post, whose presence had been expected.

Dr. E. P. Beadles, Danville, Virginia, then delivered an address of welcome on the part of the Virginia State Dental Association. He said that he was only a substitute, standing in another man's place, but that he felt himself both too short and too narrow to fill the space allotted to one who for more than half a century had been helping, by mighty efforts, to upbuild and strengthen the dental profession; one whose voice had been for many years heard in our annual meetings, and the results of whose pen were read in the dental journals all over the land; who was one of the vice-presidents of the Columbian Congress; who had been president of the Southern Dental Association, and who had filled every position, both civic and professional, with honor to himself and credit to his profession and his native State—Dr. W. W. H. Thackston—a true type of the "Old Virginia Gentleman."

He said, in the language of Dr. Crawford, I wish you, through me, to shake hands with every Virginia dentist, and to feel that our hearts are in that grasp.

We welcome you, our brothers from the North, because to know each other better will make closer friendship; you from the West, because we wish to imbibe your vim and energy; you from the Pacific slope, that you may compare the ever-rolling Atlantic with the peaceful waters of your own great ocean. We are all brothers, bone of the same bone, flesh of the same flesh. Let us join hands in the work to be done here and make this a meeting long to be remembered in the annals of dental history. "Welcome" is a magic word to Virginians; we have heard it from the time we began to lisp at our mother's knee; the "stranger within our gates" is always welcome.

Dr. Beadles then pointed out on all sides the noted historic spots, which make this region one of the most famous in American history. He concluded his address with the hope that all would find food for both mind and body, that all might depart feeling amply repaid for the trip to Old Point Comfort. Pointing to the white ribbon badge of the members of the Virginia State Association, he said, wherever you see that badge you may feel that you are free to approach and claim friendship with the wearer and that claim will not be made in vain.

In response to this address of welcome, the President of the Southern Dental Association read his annual address, which embodied a response to the address of welcome, followed by many important suggestions looking to the augmented usefulness of the Association and the advancement of the profession. The address was referred to a committee composed of Drs. J. Taft, J. Y. Crawford and B. H. Catching who, in this report at a later session, presented the following as the most salient features of the address, with the suggestion that the time of the Association could not be more profitably employed than in the discussion of these measures.

1st. The duty of our National Associations toward our State, District and Local Societies; in what way and to what extent is such aid and assistance practicable; in what way can a more intimate and closer relationship be established?

2d. The power and efficiency of State Examining Boards for good to the profession; what work can reasonably be delegated to them? In what way can they best secure the greatest good?

3d. Uniformity of laws regulating the practice of Dentistry; how can uniformity be secured? What benefits would accrue from such uniformity?

4th. To what extent is it practicable for our National Associations to influence beneficially dental education?

5th. What can our National Associations do to aid in the settlement of mooted points in practice? Is a commission for such purposes practicable?

6th. What changes should be made in clinical demonstrations to secure better results than hitherto? Can clinics be employed to advantage in all classes of dental societies?

7th. Can commissions be profitably employed for the investigation and the ascertainment of truth on assigned subjects? With what authority should such commissions be invested?

These points were presented by the committee as worthy of careful consideration.

This report was made the special order of business so near the close of the meeting that only the second and third points were discussed to any extent.

On the second point Dr. V. E. Turner said that it must be borne in mind that if there had not been a necessity for Examining Boards they would never have been created and laws would not have been enacted creating them.

The most disputed point is undoubtedly the examination of holders of diplomas.

Before the organization of the National Association of Dental Faculties, it was not possible for many of the Boards to know whether a college was "reputable" or not, and as long as there was an opening for doubts the safest plan was to examine all impartially. Laws have been passed regulating these matters. We have gone before the State Legislatures and presented the case and they have said we will give you what you want. We have but just gotten this, and shall we now say, we don't want it any longer? The examination of those holding college diplomas will not militate against progress in dentistry! Then why should we decry the laws requiring it? The Associations can do nothing in this matter. It is the affair of the Legislature of each State. Shall we go before them now and ask them to wipe out what we have just advocated as essential to our progress?

Dr. Francis Peabody: The progress of all large bodies is necessarily slow. State Examining Boards were and still are an absolute necessity, but some step must be taken to overcome the existing state of affairs in regard to the recognition of the examinations of our State Board by other Boards. I have been in practice forty years, but if I should want to locate in Mississippi I should have to be examined, if I went to Georgia I should have to go before another Board, and so of Alabama. And yet those Boards are a necessity. If the colleges graduated only competent men, there would be no necessity for Examining Boards, but there are still some colleges which work for the dollars and not for the conferring of knowledge. If a nucleus could be formed, if only one State, say one Southern State, would recognize its neighbors the idea would extend all around the borders, and great good would gradually result.

Dr. Edwards, Louisville, Ky., deprecated the assumed antagonism of the colleges and Examining Boards. If it had not been for the work of the Examining Boards the colleges would not have attained their present high standard. It is also a fact that the Examining Boards compel men to go to the colleges: they see that men are properly equipped. Before the enactment of dental laws, two-thirds of the men practicing dentistry were non-graduates. The Boards drive men to the colleges; that is the chief object and aim of State dental laws. As to the propriety of each State Board in recognizing the certificates of all other Boards, that would do very well if all Boards were appointed by properly-qualified parties; but in some of the States the Board is appointed by the Governor—by what virtue is he qualified to select the members of a Medical or Dental Examining Board? In other States this duty is entrusted to the State Dental Association.

Dr. J. Hall Moore: The State Examining Boards have done great good in elevating the standard of professional education. There is no conflict now between the National Association of Faculties and the National Board of Dental Examiners.

Dr. Peabody: As to examining men with diplomas, it is well known that some men get through College with very little education. A man should not be passed on his diploma; he should pass on his knowledge.

Dr. John S. Marshall, Chicago: If it could be so arranged that the Examining Boards could participate in the final examinations of the Colleges, placing their names on the diplomas, that would prove them reputable. A diploma signed by the Faculty and a representation from the State Boards would be a passport in any State of this Union, and the Examining Boards of the various States would recognize such a diploma. It is not necessary for the Boards to examine in all the branches which are taught in College, to prove a man's ability to practice in dentistry. I doubt if any member of the State Examining Boards could pass the final examinations of our College. A man who has been in practice a number of years gets rusty on those branches for which he has no practical use.

Dr. H. B. Noble endorsed the position taken by Dr. Marshall, and he hoped that some steps would be taken by means of which the endorsement of the State Examining Boards could be given to College diplomas.

Dr. Marshall said that his point particularly was that representatives of the State Boards should participate in the final examinations of the students in the senior classes; to see the written papers and assist in marking them; to take part in the oral examinations, and when satisfied that the students were properly qualified, to sign the diplomas as a passport to practice in any State whose Examining Board was thus represented.

Dr. Crawford: Before we can reach the solution of this question we must consider how the State Examining Boards are created, and what are the functions of the State Boards. The State Board is first a judicial body; it is also an executive body; and it is a supervisory body. As a judicial body it is the highest authority in the State on questions which pertain to its jurisdiction. The Supreme Court of the United States can not go behind the actions of the State Board. Decisions in various States have decided that. How then can an Examining Board be appointed by a State Society? They have no authority to confer judicial powers.

Dr. Peabody: The State Society of Kentucky is a chartered body for the purpose of creating the State Board, which is elected by the State Association.

Dr. Crawford: I doubt if their authority would hold good in law. A State Society may very properly recommend individuals to the Governor, but he alone can endow them with judicial powers. I have confidence in the integrity of our officials to do what is right when it is put before them.

Dr. Geo. J. Friedrichs, New Orleans, declared their State laws to be "class legislation" enacted for the benefit of the profession rather than the protection of the public. Too much has been done in the way of keeping out good men. We need to turn-about-face and get out some of those who are already in. No law is right which deprives a man of any of the functions of citizenship. I can't go into a certain State and enter into the practice of dentistry, because I have no dental college diploma. The only leg I stand on is that of M. D. That deprives me of my inherent rights and is contrary to the spirit of our institutions.

It is dangerous doctrine. I believe if I were to go before the Courts of any State, and claim my rights as a citizen of the United States, I believe I could beat any of your Dental Boards. The question has never been tried on that line.

Dr. Beadles, Danville, Va., thought the suggestion of Dr. Marshall impracticable. There are too many State Boards, too many Colleges and too many students graduating. It would be impossible to look after them all. A man who fails before the Board ought to fail to pass; he will go through if he ought to, if he comes from a college of high standards he will never fail; men have slipped through college—checked through—but the men who ought to pass the Board will pass.

Dr. R. R. Freeman: We are drifting toward a point where all can unite—the good of the whole people. There is no conflict in the truth. The men of the dental profession love to uphold and sustain the right. The leaven in the lump is working; it is in the spirit of the times, in the atmosphere. The people see we are seeking their good. The Boards will do what is right. Men who come with proper credentials will be recognized as worthy, but not all the diplomas in the world, with their red and their blue ribbons can let in the charlatan.

Dr. Friedrichs: Dr. Freeman speaks for the people; I speak for the dentist. The man who has his diploma, or his board certificate must be able to go from State to State without further expense or trouble. A man who has exhausted his field must be able to seek another.

On the third point, Dr. Donelly, Washington, D. C., spoke of the great necessity of harmonizing and unifying the State Dental laws. As they now stand there are gross incongruities and inconsistencies. A fixed educational standard should be established. A diploma or State certificate of the attainment of this standard would bring all in practical unity. Let a law be drafted which shall meet the endorsement of the National Association of Faculties, of the National Board of Examiners, of the American Dental Association, or the Southern Association. I think the profession would all unite on such a law. Then carry it before the Legislature of one State after another and get it adopted. It would be a disadvantage to any State not to adopt a dental law having such endorsement. Dr. Turner said we could not ask for a change in the law we had demanded; I say we could, upon the ground that the law had accomplished what it was designed for, and in the natural course of progress, we now need something different. It puts a man at a great disadvantage to have to contend with this great diversity of laws. (Dr. Donelly referred to a decision of the United States Supreme Court in a West Virginia case, published in the *Dental Cosmos* late in 1890 or early in 1891).

Dr. Francis Peabody spoke on the same line. He hoped that through the present meeting of the different Associations something would be developed that can be presented to the different State Legislatures that will prove satisfactory to the Colleges, to the Examining Boards and to the profession.

In this connection, Dr. R. R. Freeman offered the following resolution:

*Resolved*, That the Southern Dental Association, with a view to unity, harmony and concerted action, appoint a special committee on Dental Legislation, to represent it in any movement in which its co-operation may be sought or its "good offices" accepted for the improvement and unification of dental laws.

The following committee was appointed: Drs. R. R. Freeman, M. F. Finley, J. Y. Crawford.*

On the fifth point—The consideration of mooted points in practice.

Dr. J. Y. Crawford said he had been disappointed in not having been able to carry out a plan he had designed for the settlement of our mooted questions. He had understood that a paper would be presented on the methods of treating and filling root-canals. He had prepared a considerable number of teeth, properly imbedded in plaster, and he had hoped to have had the root-canals filled by the individual advocating certain methods; the teeth to be then submitted to a commission who would make

*This subject was also very fully discussed in the American Dental Association, and the following committee on Unification of Dental Laws appointed: Drs. B. Holly Smith, A. W. Harlan and J. Y. Crawford, to confer with similar committees from other Associations.

sections of the roots and ascertain definitely which method was best calculated to fill the requirements of the case. The paper had not been presented, however, and his scheme had fallen through.

DR. JNO. S. MARSHALL said that for a commission to take into consideration "mooted points in practice," it would be necessary for them to review the scientific work presented; to go over experiments, study methods, etc. This would require both time and money, and unless the Association was in position to vote a fund for that purpose, the plan would fall through. In the American Dental Association money has on different occasions been voted to various sections to work out different lines of special scientific thought, but it is necessary to have money to carry this out successfully. He added that he would gladly vote for money to be spent in such scientific investigations.

DR. V. E. TURNER suggested that a committee be appointed to take this matter in hand and report at the next meeting. This committee can suggest the amount considered adequate for this purpose, and if the Association can raise it, we will have the advantage of thorough scientific investigation of certain questions, which will be reliable authority in the conclusions reached.

On motion of Dr. Jno. S. Marshall:

*Resolved*, That a committee composed of three members of this Association be appointed to serve as many as two, three and four years, to take under consideration and examination such mooted questions in practice as may be referred to it by the Association and that they shall make an annual report to the Association with such conclusions and suggestion as grow out of their individual and collective work.

Committee appointed by the Chair as follows: John S Marshall, H. B. Noble, R. R. Freeman.

In the American Dental Association a commission of five—Drs. C. N. Peirce, J. S. Marshall, E. C. Kirk, M. L. Rhein and J. Taft were appointed to investigate as thoroughly as possible, etiology, pathology and treatment of Pyorrhœa Alveolaris, and report at the next meeting. This commission was authorized to draw on the treasury of the Association for a sum not exceeding seventy-five dollars to meet the expenses of this work.

Dr. I. N. Carr, Tarboro, N. C., read a paper on "Dental Education." He emphasized the necessity for thorough preliminary education for the dental student, superior qualifications being now demanded in every department of human industry. Excellence is the only warrant of success. The dentist of to-day must have a good college education, mechanical ingenuity, and varied and substantial accomplishments for his social needs. The dental colleges have all the advantages and facilities for teaching the advanced branches necessary in professional education, but they have too many students to allow them to teach the elementary fundamental branches. They can not teach anatomy perfectly, for with two hundred students in the class only those well up in front can see what is being demonstrated. In the dissecting room he must learn what he can, practically unaided.

In this busy age reading with a preceptor is time lost; the able practitioner is too busy or he is rusty on some branches and he can furnish no dissecting material. A thorough course in a good preparatory school is of inestimable benefit to the dental student. Dr. Carr places a high estimate on the value of proper reading, selecting only the works of the best authors. Books create a social world for us; in them we find congenial fellowship; they feed the mind and by them we grow into larger stature. They add the senses of others to our own and we gain a clearer eyesight, a keener touch, more acute hearing. Life is multiplied by them. They open the highways of thought along which we are borne on distant journeys. They respond to our aspirations more heartily than our truest friends. They interpret our thoughts and make clear the dim conceptions of our own minds, perfect our half formed ideas, assure our hesitancy and relieve our doubts. We should read, not merely to get information, but to go a step higher and become thinkers.

Among the requirements of the successful, cultured dentist, Dr. Carr enumerates good manners, unaffected politeness to all, kind and gentle yet firm behavior with patients, placing himself in perfect harmony with his surroundings.

In regard to the duty of the dentist in instructing the general public, Dr. Carr suggests that the State Societies, or better still, the Southern Dental Association issue a suitable pamphlet, and that a publishing fund be created for this purpose.

In connection with points two and three of the above report, it may be stated here that able papers on Dental Legislation were read by Drs. H. B. Noble and A. W. Sweeney, of Washington, D. C. Both papers were in the line of condemnation of the State laws which require the re-examination of College graduates —laws that in the view of Dr. Noble "seem to be a slur on College and University work." If necessary to supervise the work of the Colleges, it shonld be done by properly-delegated authority, so that a diploma shall represent truly such a course of training and knowledge of dentistry as shall entitle its holder to practice, without further examination, in any location he may select—subject always to the laws against malpractice. A dishonest or dishonorable lawyer can be debarred from the practice of law, so, also, the dental laws should be so framed as to debar from the practice of dentistry the man who is unworthy or incompetent. The dental law of Connecticut is nearer the mark in that respect than any other State law as far as known. Under certain conditions the State Examining Board can cancel the certificate conferring the right to practice.

The laws should be so framed as to reach the advertising quack rather than to turn down some boy-graduate not quite up in book-examinations, or to catch some old graduate who is getting rusty on anatomy or chemistry. Examining Boards should handle the student before he gets his diploma, so that the diploma would represent both the Board and the College. If it believed that the Colleges are not educating their students up to the proper standpoint, then committees should be appointed to investigate and report the Colleges that are not up to the mark.

Every College should have a representative in the Examining Board of its State, to serve as a friendly connecting link, promoting harmony and mutual interest between the Boards and the Colleges. Dr. Noble suggested that committees be appointed from the Southern and the American Associations to act and confer with the National Board of Faculties and the National Board of Dental Examiners, and recommend such changes as will tend to harmonize the dental laws and promote dental education.

Dr. Sweeney's paper also dealt with needed reforms in dental legislation. Dr. Sweeney can not find in all that has been said on the subject "one particle of argument to sustain the position of those who try to maintain that no college degree or State certificate shall be accepted in lieu of examination, and that even though re-examined in one State in order to practice, it must be done *over again* in another, if the individual should see fit to move." He is not opposed to dental laws, provided they are designed to accomplish only proper results. He hopes to see laws which will not "wink at" the prosperous and arrogant quack while they are made to seize upon and terrify the young and struggling graduate who has just parted with his scanty means in the effort to learn how to earn an honest living, and are held up as a menace to the practitioner from another State or country, whose ability may be great and his intentions most honorable—laws which, if they can not be made "retroactive," can be made more clearly to define what constitutes malpractice in dentistry, and impose some wholesome restriction upon the reckless abuse of anæsthetics, which is inflicting incalculable injury, pandering to false sentiment, preventing men from seeking competent dental service and perpetuating the ignorant idea of the past generation, that the dentist is merely a tooth-puller and a maker of "false teeth."

The lack of judicious provision in our dental laws for the suppression and prevention of practices injurious to the public, constitutes their most conspicuous weakness, while the evidences which they disclose of efforts on the part of their framers to incorporate in them monopolistic tendencies, must be justly regarded as their reproach. The "protective system" is unethical, narrow, and utterly indefensible when applied to the regulation of professional practice, and a great part of the adverse criticism noted against the dental laws can be traced to obvious efforts on the part of their framers to embody in them, provisions which tend to give to dentists already in practice an advantage over those yet to enter. So long as the most unblushing charlatans are permitted to flourish in all large cities where dental laws are in force, while the law is ever held up as a menace to the graduate about to start in practice, or the competent and well-meaning practitioner from another State, just so long will recruits be furnished to the ranks of those who denounce dental legislation as a delusion, if nothing worse.

Dr. Sweeney cited as precedents the laws of Connecticut and New Mexico, which contain provisions for *working* as well as for granting licenses to practice, as contemplating a more practical effort towards the protection of the public than those which simply demand the examination of those commencing practice. A requirement barren of practical results in the direction of protection. Dental laws should be framed to protect the interests, not of the dentists of the state, not of those who frame the law, not of the colleges, but of the people of the State.

Dr. Sweeney closed his essay with the prediction that "before the first sunrise of the coming century, the honesty, the true professional spirit, and the sound common sense of our colleagues, the world over will have swept aside every clumsy and ill-advised measure calculated to retard our healthy growth, and dentistry will stand in the vanguard of the liberal professions, dispensing its blessings with an increased and ever-increasing bounty."

Dr. C. N. Pierce being called upon to open the discussion of these papers said that he was compelled to dissent from much in both papers. Why have these laws been enacted? There must have been a reason—some foundation for them—that foundation is found in the multiplicity of dental colleges. With perhaps five professors and ten demonstrators in each college, giving their services without compensation, what is the inference? They must have students and they must hold out special inducements. They say, "come to us and we will give you an education and a degree. You shall get your diploma as soon as possible." But some could not get enough students on those terms and they said virtually, we will give you the diploma without the education, and that is the foundation of our dental laws. Our present dental laws are in the line of progress; gradually they are bringing up to ideal conditions. The schools should be the creators of scholars, not grantors of degrees. In a class of two or three hundred students, it is not possible that all shall be equally studious; some are sharp, some get through and really know but little; it can not be avoided; it is the natural result of the conditions. That a student who is not qualified sometimes gets his degree is not to be wondered at; the only wonder is that there are not more! The disposition now is not only to raise the standard of preliminary education but to extend the time of study, and to make the student so proficient that he will not fear to go before any Board.

Dr. B. H. Catching said that if it were possible to do away with the Examining Boards it would be a bad thing for the colleges, for they would lose half their patronage; they go hand in hand. A first-class Dental College graduate has no hesitancy in going before any State Board. They need have no fear if they are competent. The examinations of the State Board are on practical lines; as a rule they are fair, easy and practical. Blot out the Examining Boards and you deprive the colleges of half their patronage.

Dr. R. R. Freeman: The millennium has not yet arrived. There is an unrest, an unsatisfied condition. Men must learn that to do right is the means to prosperity; we must have clean hands and pure hearts. Dr. Freeman thought the method adopted in the legal profession would be good in dentistry. A man shows what he knows before the bar; he is not examined, but if he does not give evidence of being all he claims, he is disbarred.

Dr. H. J. McKellops: I have not language to express what I feel. I sprang from nothing. I educated myself. I am proud of my profession, but I am not proud of our colleges or of our laws. We want laws to govern the colleges and make the professors teach the students. There are now forty schools and if a man can't pass in one he passes in another. If he can't pass the Board in one State he passes in another; but a man who has practiced forty years in one State can't go and practice in another. It is not a good law. It is not so in the legal profession. I go the world over, and I watch these things carefully. Dr. McKellops then described the existing condition of things in England and the causes leading to the present condition.

Dr. W. C. Barrett explained at length the workings of the University of the State of New York, with its Board of Regents, the methods of the Regents examinations, the relations of all educational institutions in the State of New York, including law schools, medical schools, and most recently of the dental schools to the university. It is not a teaching institution but a supervisory body, it grants charters and revokes charters. Through its examinations only are students admitted to the higher educational institutions. It established an unvarying standaid from which there is no appeal. A student must have attained a certain standing in preliminary education before he can matriculate in any professional school. He cannot matriculate whether in law, in medicine, in divinity or in dentistry without a Regents' certificate. And when he has graduated he has no diploma; he is not licensed to practice until he has been examined by a Board appointed by the Regents. If the report of the Board is satisfactory to the Regents then he receives his license. Only within the past year has dentistry been admitted on the same standing as the other profession. Dr. Barrett deems this a long step in advance. Charters are granted only by the Board of Regents, and no medical college can be chartered until it has $500,000 actually raised and deposited as an endownment fund.

Dr. Frank Abbott further elaborated the scheme of the University of the State of New York and the advantages accruing to the dental profession in that State by the alliance of the dental schools with the university. He said, if we lose in students we shall gain in self respect.

Dr. T. B. Welch illustrated the workings of the Board of Regents by the following incident which occurred many years ago: A young man desiring to practice medicine applied for a certificate from the Board of Regents. He was a graduate of a civic course college and was well read in medicine, though he had only been in a medical college four months, and in a medical office six months. He frankly stated these facts to the Board. Said he, "They took us in hand and said, 'Now boys, we have no written questions; we are not going to put on the screws, but we will have a general conversation. We do not ask you where you got your information, or how long you were in acquiring it, we shall soon find out if you are competent. If so, we will give you a certificate; if not, whether you have studied one year or ten years, you will not get it!'"

Dr. Welch also explained the requirements for permission to practice dentistry in England, and the steps necessary in obtaining a degree from the Medical Council. He said you must go to the hospital and pass two years there just as though you knew absolutely nothing about dentistry.

Dr. McKellops said that England was only acting towards American dentists exactly as one State here does towards another and he did not see why all this howl should be raised about it. There is no little feeling of jealousy in it, in arraying one State against another State, one college against another college, but simply the desire to see that men are really competent to do what they profess to do. He thought a committee ought to be appointed to visit our own colleges and look into their manner of conducting business.

Dr. W. H. Morgan thought it looked as though there was a good deal of politics mixed up in the Board of Regents business. He did not see how anything but a corrupt stream could flow from a corrupt fountain, and judging Albany by the reports in the New York newspapers it would be hard to find anything worse for wire-pulling and chicanery.

Dr. J. Y. Crawford thought the discussion had wandered far from the two papers read. Dr. Noble's paper was unique in the solution of the difficulties offered, but it had one weak point. The presence of a member of a College Faculty or a State Examining Board would vitiate the Board and deprive it of its functions; this was the decision of the Supreme Court. He himself had been in his own State Examining Board, but now that he had been elected to a College Faculty he had laid down the other position; he could not serve in a dual capacity. He was disqualified by the action of the Supreme Court.

There is but one rational, just and philosophical solution of this question. We have in each State a Board legally qualified to pass upon the reputability of the dental institutions of this country.

If it is proved to the satisfaction of the Board that they have complied with every requirement, then the diploma of that institution should guarantee to its holder the right to practice dentistry in any State in this country. That should put him in *statu quo* in every State. That is the only solution of this question. An additional feature that is required has been placed on the statute books of one or two of the States, and that is, that when a man is proved guilty of unprofessional conduct he can be deprived of his license. If it is right to disbar a lawyer if he is guilty of falsifying his word or defacing the records, then it is right to revoke the privilege of practicing dentistry if a man is guilty of unprofessional actions. This is the way to put an end to charlatanism.

DR. OTTOFY: It is impossible for Congress to supply the remedy in this case. The States are sovereign and each State must improve its own law. The bad laws that we now have, have been copied one from another beginning with the oldest.

DR. EDWARDS spoke in approbation of the Board of Regents of New York and hoped such a board of control would be secured for Illinois.

DR. SWEENEY, in closing the discussion of his essay, said he did not see why it should be deemed impossible to attain such a degree of excellence that a license granted by one Board should be recognized by another. All that is necessary is to set the standard high enough.

DR. NOBLE, closing the discussion in his paper, said in reply to Dr. Crawford, that all that was neccessary to enable college professors to hold places on the State Examining Boards was to have laws enacted to that effect. He had been on an Examining Board with a teacher from a college and found him able to supply the Board with much valuable information. It should be a matter of pride on the part of the colleges to obtain representation in the Boards, to see that their work is up to the standard of excellence of their fellowmen. If such a law is demanded it can be secured. Subject passed.

[*To be continued.*]

